# Perceived benefits and barriers of medical doctors regarding electronic medical record systems in an Indian private-sector healthcare facility

**DOI:** 10.1186/s12913-025-12877-5

**Published:** 2025-05-19

**Authors:** Hager Saleh, Cecilia Stålsby Lundborg, Megha Sharma

**Affiliations:** 1 Department of Global Public Health, Health Systems and Policy, Karolinska Institutet, Stockholm, 17177 Sweden; 2 Department of Pharmacology, Ruxmaniben Deepchand Gardi Medical College, Surasa, Ujjain, 456006 India

**Keywords:** Electronic Medical Record (EMR), Electronic Medical Record System (EMRS), Private-sector healthcare facility, Implementation barriers and benefits, Medical Doctors, India

## Abstract

**Background:**

Electronic medical record systems (EMRS) have transformed healthcare by improving quality, efficiency, and safety through centralized patient data and streamlined workflows. Challenges such as budget constraints and staff resistance hinder its adoption, particularly in resource-limited settings like India; however, they have not been investigated thoroughly. The objective of our study is to explore the benefits and barriers perceived by medical doctors in implementing EMRS in an Indian private-sector hospital.

**Methods:**

A cross-sectional study was conducted at a private hospital in a rural area of the Ujjain district, Madhya Pradesh, India. All 130 doctors pursuing postgraduate studies were invited to participate in person using a convenience sampling method. Responses from 105 doctors were received using a self-administered questionnaire. The survey captured participants’ attitudes, perceived benefits, and barriers to EMRS implementation in the facility. Descriptive statistics were used to analyse the data.

**Results:**

Of the respondents, 93% expressed a desire for EMRS implementation. Identified barriers included financial constraints, insufficient infrastructure, staffing shortages, and technological challenges, such as unreliable internet access. Participants highlighted the anticipated benefits, such as improved data accessibility, enhanced operational efficiency, and preferences for digitizing lab reports and e-prescriptions.

**Conclusion:**

Exploring and addressing the financial, organizational, and technological barriers as perceived by the participants are crucial steps to facilitate EMRS implementation in healthcare facilities. Larger in-depth studies are necessary to develop tailored strategies for overcoming these challenges in similar settings.

**Supplementary Information:**

The online version contains supplementary material available at 10.1186/s12913-025-12877-5.

## Background

An electronic medical record system (EMRS) is a digital repository of the patient’s health information [1, 2]. It can include medical history, diagnoses, medications, treatment plans, allergies, and test results, resulting in a large database that enables real-time access for healthcare professionals [1, 2]. EMRS enhance healthcare quality, efficiency, and patient safety [3, 4] by supporting informed decision-making, streamlining administrative tasks, and improving communication among healthcare teams [1, 5]. It provides alerts for allergies, drug interactions and supports telemedicine and remote healthcare access, expanding healthcare services to underserved areas [2, 4, 6–8]. However, successful implementation requires acceptance and readiness within healthcare facilities [9].

The adoption of EMRS is complex and multifaceted, influenced by interconnected factors such as the organization’s infrastructure, technological readiness, regulatory compliance and healthcare providers’ training [9]. Although there is substantial research on EMRS implementation dynamics in high-income countries (HICs) [9–11], a critical gap remains in understanding how these systems function within the unique context of lower-middle-income countries (LMICs).

Health Management Information Systems (HMIS) serve as a foundation for digital health tools, supporting the collection and use of health data [12, 13]. India is transitioning from HMIS to comprehensive EMRS adoption through initiatives like the Ayushman Bharat Digital Mission, which integrates data from public and private healthcare facilities and streamlines reimbursement processes [14]. Digital interventions, such as Co-WIN and U-WIN, demonstrate EMRS potential in resource-limited settings [15]. However, infrastructure gaps, resource disparities, and workforce limitations remain challenges, highlighting the need for focused research [16]. The challenges faced by LMICs are different from those faced by HICs, and India presents a unique scenario with factors significantly affecting EMRS adoption [17].

India’s healthcare is divided into the public and private sectors, with a diverse landscape, ranging from small 10-bed clinics to 5,000-bed hospitals [18–20]. The large population stimulates challenges such as high patient loads, workforce shortages, and antimicrobial resistance, creating obstacles for EMRS adoption [18–20]. Moreover, private facilities, the most common healthcare providers, often operate with constrained resources and regulations [21]. Data management practices also vary significantly between public and private settings, leading to gaps in uniformity and comprehensiveness [16]. Understanding these variations is crucial for developing standardized EMRS frameworks in resource-limited environments.

Prelaunch assessments are vital to identifying challenges before full-scale EMRS implementation [22, 23]. Healthcare professionals’ readiness and competency play a critical role in determining implementation success [24]. Studies from HICs provide valuable insights. A study in a tertiary care setting in the Western Sydney local health district found that, before EMRS launch, clinicians anticipated improved data completeness but expressed concerns regarding increased workload [23]. Post-launch, benefits in data efficiency were observed, though concerns remained about workload management, system functionality, and staff acceptance. These findings underscore the importance of ongoing support and refinement in EMRS implementation [23].

In India, a study at primary health centres (PHCs) identified budget constraints, lack of IT staff, and perceptions of EMRS as time-consuming as major barriers [25]. Another case study in an Indian hospital found that stakeholders’ engagement, iterative system design, and staff incentives were key to successful adoption [26]. A global literature review identified eight categories of barriers to EMRS adoption: financial, technical, time, psychological, social, legal, organizational, and change process [27]. In Saudi Arabia, a study found strong leadership and change management were crucial for overcoming implementation barriers, reinforcing that EMRS adoption is a socio-technical transformation, not just a technical project, requiring strong leadership, effective communication, and continuous training [28].

Understanding the complexities of EMRS implementation is essential for developing context-specific strategies that address the distinct challenges of healthcare systems in LMICs. Recent studies have emphasized the need for tailored approaches in EMRS in LMICs [10, 29, 30]. For example, a comparative analysis found that EMRS adoption in HICs is often supported by robust infrastructure, advanced healthcare systems, and comprehensive policies [10]. In contrast, LMICs face challenges such as inadequate infrastructure, financial constraints, and insufficient regulatory frameworks [10]. Additionally, healthcare providers’ preparedness and acceptance remain underexplored, emphasizing the need for context-specific approaches in LMICs.

Despite these valuable insights, a deeper understanding of hospitals’ staff preparedness, perceived benefits, and challenges is crucial for successful adoption [16, 26, 31]. Thus, this study aims to address this critical gap by investigating the benefits and barriers associated with EMRS implementation among medical doctors in a private-sector hospital in Central India. Exploring these factors will contribute to a body of knowledge that can guide the integration of tailored EMRS, with implications for both healthcare practice and policy development.

## Methods

### Study setting and study participants

This study was conducted at a private-sector hospital with 800 beds, situated in a rural setting in Central India. The hospital provides tertiary-level medical care and is affiliated with a medical college within the Ujjain district of Madhya Pradesh state [32]. It has served an average of 1760 outpatients and 157 inpatients per day in the year 2024. The medical records are maintained using paper files.

In India, postgraduate medical education offers two types of 3-year specialized programs for medical doctors (MBBS graduates, i.e. Bachelor of Medicine and Bachelor of Surgery). It includes: (1) the Doctor of Medicine (MD), which offers training in medical specialization without surgery and (2) the Master of Surgery (MS), which focuses on surgical specialities. There were no new admissions in these postgraduate programs during the COVID-19 pandemic period, resulting in no participants from the second year. Therefore, the study participants were medical doctors enrolled in the first or third year of one of these postgraduate programs. These doctors were responsible for providing clinical services and patient record documentation.

### Method of data collection

The data were collected between May and July 2023 using the convenience sampling method. Following the all-inclusive method, all medical doctors enrolled on postgraduate programs during the study period were invited to participate in any of the multiple sessions conducted for physical data collection. Each session comprised an explanation of the study goals, its ethical foundations and a clarification that the data will be presented only at the group level, followed by responding to the printed questionnaire. Out of the 130 invited doctors, 105 participated and filled out the questionnaires in nine sessions. The surveys were filled out in 20–25 min. Additionally, participants were encouraged to ask questions if they needed further explanation or clarification on the topic.

### Data collection tool

The development of the questionnaire was a systematic process guided by expert opinions and relevant existing literature [33–37] (see supplementary file). Additionally, the questionnaire was pretested with a sample of ten participants at different time points (not included in this manuscript). During this pretest, participants provided feedback on the clarity of two questions and instructions for filling out the questionnaire. Based on their feedback, minor adjustments were made to refine the question’s wording, ensuring the survey’s clarity and validity of the questions. Reliability was ensured through test-retest reliability by administering the questionnaire to four participants from the pretesting phase at two different time points, with a one-month interval, to check for consistency and stability in their responses. Cohen’s Kappa [38] was calculated to determine the percentage of agreement between the test and retest results, yielding a Kappa value between 0.85 and 0.90, indicating a strong agreement.

The structured questionnaire encompassed various sections, each designed to capture specific benefits and barriers to the implementation of EMRS in the inpatient departments of the hospital. These sections included the following information:

- *Demographic information*: sex, department, and qualifications.
- *Attitudes towards EMRS*: Capturing respondents’ attitudes and perceptions regarding EMRS adoption, including any concerns or expectations they might hold.
- *Perceived benefits of EMRS*: Exploring respondents’ expectations regarding the benefits of EMRS, including improved patient care, enhanced clinical efficiency, and more effective data management.
- *Perceived barriers to EMRS*: Investigating potential challenges and obstacles associated with EMR implementation, including financial constraints, infrastructure limitations, and resistance to change.

The perceived benefits and barriers were expressed through categorical responses, grouped into distinct categories for barriers: “Major Barrier,” “Barrier but possible to Manage,” and “Not Applicable”; and for benefits: “Major Benefit,” “Minor Benefit,” and “This is not a Benefit but a Barrier”. Each section, except the demographic section, included an open space and open-ended questions to record additional reflections of the participants.

### **Data analysis**

The data were entered into the Epi Info software version 3.1 and subsequently exported to an Excel spreadsheet to check its completeness. The data were then imported into STATA version 18.0 for analysis. Cohen’s Kappa was calculated to determine the reliability of the questionnaire [38]. Descriptive statistics, specifically frequencies, were utilized to identify underlying patterns and trends in the data. To examine associations and determine the significance of relationships, the chi-square test was employed, allowing us to identify significant connections and factors that influence the adoption and perceptions of EMRS. Additionally, responses to open-ended questions were grouped into key categories and quantified to summarize participants’ perspectives on EMRS.

## Results

### Sociodemographic characteristics

The participants’ sex was evenly distributed (male-51%) across the 105 respondents (Table 1). The Anesthesia department had the highest representation (17%), followed by Surgery (15%), Obstetrics and Gynecology (14%), and Medicine (13%). Most of the participants (53%) were in their first year of postgraduate education.

**Table 1:** Sociodemographic characteristics of the respondents in an Indian private-sector hospital

Characteristics	Total *N* = 105; n (%)*
Sex	
Female	51 (49)
Male	54 (51)
Departments	
Medicine	14 (13)
Obstetrics and Gynecology	15 (14)
Surgery	16 (15)
Orthopedics	12 (12)
Ear, nose, and throat	8 (8)
Ophthalmology	11 (10)
Tuberculosis	5 (5)
Dermatology	6 (6)
Anesthesia	18 (17)
Years of postgraduate specialization	
First-year	56 (53)
Third year	49 (47)

*The percentages are rounded off to the nearest number

### Attitudes towards the implementation of EMRS

**Fig. 1:**
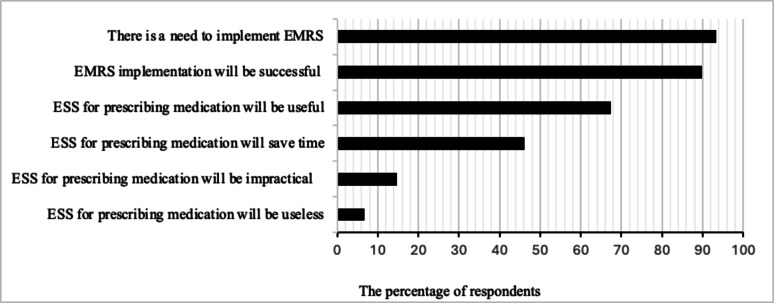
Attitudes of respondents towards the use of EMRS (*N* = 105). Abbreviations: EES: Electronic support system, EMRS: Electronic medical record systems

93% of the respondents agreed with the need to implement EMRS in the inpatient departments of their facility (Fig. 1). 68% of participants thought that a prescription electronic support system (ESS) would be beneficial, while 14% and 7% stated it would be impractical and useless, respectively. On the other hand, attitudes toward the potential time-saving advantages of EMRS were mixed, with 46% thinking it would save time. A statistically significant association was found between the year of postgraduate specialization and the belief that the ESS for prescribing medication would be useful (Pearson χ²= 7.5255, *p* = 0.006), where 68% of third-year specialization doctors believed in the usefulness of the EES.

### Awareness of the data recording and analysis to assess healthcare quality in the facility

**Fig. 2:**
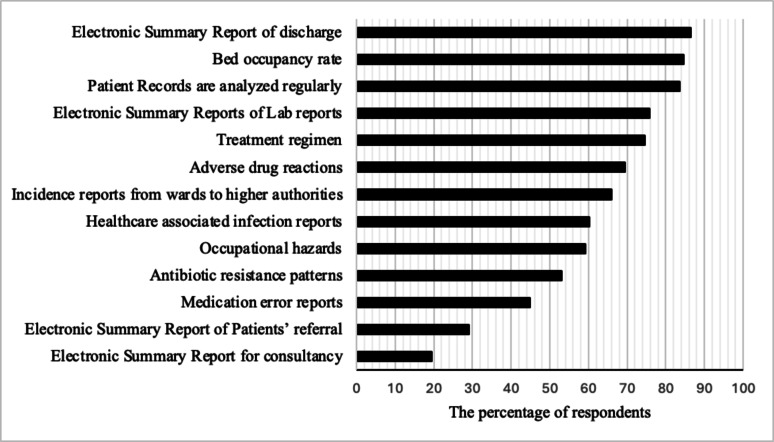
Participants’ awareness of the reports collected, analysed, and presented in the inpatient departments of an Indian private-sector hospital

The respondents’ awareness of the types of data that are frequently recorded and analyzed in the study hospital is presented in Fig. 2. Forty-five percent of the participants were aware of medication error reports, 75% were aware that treatment regimen reports were present, 69% said they were aware of adverse drug reaction reports, and 53% of the respondents were familiar with reports on antibiotic resistance patterns. Additionally, 86% indicated the presence of electronic discharge report summaries, and 76% of the respondents mentioned the existence of electronic summary reports for lab results.

### Perceived barriers to EMRS implementation

The following are the main perceived challenges and barriers associated with the implementation of EMRS (Table 2);

**Table 2:** Perceived barriers to EMRS implementation in an Indian private-sector hospital

Barriers	Major barrier *n* (%)	Possible to manage *n* (%)	Not applicable *n* (%)	Total *N*
Financial components				
Lack of funds	43 (44)	47 (48)	9 (9)	99
Capital investment	35 (37)	47 (47.5)	12 (13)	94
Organizational components				
Time management*	27 (29)	62 (65)	6 (6)	95
Lack of infrastructure	47 (47)	45 (44)	9 (9)	101
Lack of staff	57 (56)	36 (35)	9 (9)	102
Staff resistance**	51 (52)	33 (33)	15 (15)	99
Lack of IT personnel	59 (58)	35 (35)	7 (7)	101
Staff competency	51 (51)	41 (41)	8 (8)	100
Lack of staff coordination	47 (48)	46 (46)	6 (6)	99
Possible loss of productivity	34 (34)	50 (50.5)	15 (15)	99
Changeover of information	30 (31)	50 (51.5)	17 (17.5)	97
Lack of a trainer on EMR	44 (44.5)	47 (47.5)	8 (9)	99
Lack of management support	47 (46)	44 (43)	11 (11)	102
Legal issues				
Concerns for confidentiality breaches	31 (31)	55 (55)	14 (14)	100
Regulations of electronic signatures	29 (29)	59 (60)	11 (11)	99
Legal acceptability of EMRS	25 (26)	52 (54)	19 (20)	96
Technological issues				
Finding a convenient system	39 (39)	54 (53)	8 (8)	101
Concerns about the EMR system updates	22 (22)	57 (56.5)	22 (22)	101
Unreliable Internet access	42 (42)	44 (44)	14 (14)	100
Incompatibilities of Software/hardware	37 (37)	53 (53)	10 (10)	100
Training about EMRS	39 (39)	55 (55)	6 (6)	100

*Pearson chi^2^ (χ² test), Pearson χ²= 6.1662, *P* = 0.046. **Pearson chi^2^ (χ² test), Pearson χ²= 26.3529 *P* = 0.049. The percentages are rounded off to the nearest number

- *Financial and Legal Concerns*: A significant segment (44%) of respondents pointed out that insufficient funds could pose a major obstacle to EMRS implementation. Additionally, 31% expressed worries regarding potential breaches of confidentiality, whereas 29% raised concerns about regulatory obstacles related to the electronic signatures in the EMRS.
- *Organizational Challenges*: The organizational aspect revealed multifaceted challenges. The shortage of staffing was pointed out by more than half of the respondents (56%), along with resistance from existing staff members. Moreover, the absence of dedicated information technology (IT) personnel was cited as a major barrier by 58% of the respondents. Staff competency was considered crucial for seamless EMRS utilization; therefore, it was also considered a major barrier by half of the respondents (51%). A total of 47% highlighted the lack of necessary infrastructure as a significant barrier. The lack of coordination within the healthcare system was emphasized by 48%, perceiving it as a major barrier. Furthermore, the absence of management support was acknowledged as a significant barrier by 46% of the respondents.
- *Technological challenges*: The necessity of reliable internet access to support the efficient utilization of EMRS was underlined by 42% of the respondents. Additionally, 39% of the participants identified difficulties in selecting an appropriate system for the hospital and the availability of a systematic training program for the staff as significant obstacles to EMRS implementation.

The Pearson chi-square statistic of *p* > 0.05 indicates a statistically significant association between time management as a barrier and the successful implementation of EMR systems (Table 2). Additionally, a statistically significant association was observed between the affiliated departments of respondents and staff resistance (Pearson χ²= 6.1662, *p* = 0.046), indicating that different departments may have varying levels of readiness and willingness to support EMRS technology. Staff resistance was identified as a major barrier by 72% of respondents from the medicine department and 57% from both the obstetrics and gynecology department and the surgery department.

In response to the open-ended question regarding additional barriers to digitalizing medical records in their healthcare facility. Participants expressed concerns about the lack of technological knowledge among the staff and the lack of knowledge and awareness about EMRS, this was reflected by Participant-79 (P-79), “*Lack of technological knowledge exists among staff*” and P-52, “*Lack of time and awareness is there*”. They also expressed concerns about the time required to initiate and maintain EMRS, which will be a significant challenge, as noted by P-102, “A l*ot of time will be required initially to start EMR and to maintain it*”. Additionally, the lack of training among nursing staff was reported as a factor increasing the rigidity in completing their daily tasks, and placing an additional workload on the duty doctors, “*Nursing staff is never trained for any digital work*,* their rigidity to do any work always create a burden on the duty doctor*” (P-95). Finally, staff dissatisfaction was stated as a barrier to the successful implementation of EMRS; P-80 wrote, “*Unsatisfied and unqualified staff is a barrier*”.

### Perceived benefits of the EMRS implementation

Seventy-nine percent of respondents anticipated that EMRS could revolutionize the accessibility of clinical data and enable convenient retrieval of patient information from any location at any time. Operational efficiency was another perceived advantage, with 79% of the participants anticipating that EMRS would improve healthcare processes through digital record-keeping. Furthermore, 54% of the participants anticipated EMRS to empower staff and increase their satisfaction, potentially making the facility more attractive for future employees (Table 3).

**Table 3:** Perceived benefits of EMRS implementation in an Indian private-sector hospital

Benefits	Major Benefit *n* (%)*	Minor Benefit *n* (%)*	This is a Barrier *n* (%)*	Total *N*
1. Anyone from anywhere, at any time, can access the clinical data	77 (79)	11 (11)	10 (10)	98
2. Management control will be easier	83 (80.5)	14 (13.5)	6 (6)	103
3. Quality of monitoring will be easy	81 (80)	14 (14)	6 (6)	101
4. Enhanced efficiency due to digital records	78 (79)	19 (19)	2 (2)	99
5. Staff will be empowered, and staff satisfaction will be obtained	54 (54)	40 (40)	6 (6)	100
6. An attractive job feature when recruiting new staff	50 (49.5)	39 (39)	12 (12)	101
7. The information entered at one point can be seen on other screens at different places; this will save time, i.e., faster and more accurate billing with integrated data systems.	80 (78)	19 (18.5)	4 (4)	103
8. Improved regulations of treatment compliance	63 (62)	35 (34)	4 (4)	102
9. Ability to electronically exchange data with other providers, hospitals, medical offices, labs, and pharmacies: faster exchange	79 (77.5)	18 (18)	5 (5)	102
10. Cost savings and time saving	67 (66)	24 (24)	10 (10)	101
11. Patient safety (minimum medical errors due to handwriting errors, such as, due to like and sound like errors)	70 (67)	26 (25.5)	6 (6)	102
12. Treatment planning can be improved as all information will be available at one point	77 (75)	23 (22)	3 (3)	103
13. Improved communication within the facility: among staff between shifts, labs, and various wards	81 (79.5)	18 (18)	3 (17.5)	102

* Percentages are rounded to the nearest number

In response to the open-ended question about any further anticipated benefits of using EMRS, a variety of advantages were expressed by participants (*n* = 60, 57%). The responses, mostly provided in a single sentence, were grouped into categories, as shown in Table 4.

**Table 4:** Categorized responses to open-ended questions about the anticipated benefits of EMRS from participants

Category	Total *N* = 60; n (%)
Accessibility and retrieval of medical records	21 (35)
Medication management and planning	11 (18)
Efficiency and timesaving	11 (18)
Paperless and cost-effectiveness	5 (8)
Improvement in the healthcare system	5 (8)
Legal benefits	2 (3)

#### Accessibility and retrieval of records

Participants noted that EMRS offers significant benefits in terms of accessing and retrieving medical records. They emphasized the ease of checking old records and patient history, which facilitates long-term record-keeping for future consultations. The system’s ability to provide quick and easy access to patient history records was seen as enhancing efficiency and data retrieval, particularly for handling large amounts of information and conducting long-term information gathering. This was reflected by P-61, “*It [EMRS] will be much easier to gather information from about the past 10–15 years*.”, and P-30, “*It will give easy access to medical records of patients*”.

#### Medication management and planning

The respondents anticipated that EMRS implementation could assist and promote a better understanding of prescriptions, promoting rational medicine use and ensuring proper medication prescribing, a facet that is currently lacking. P-23 wrote, *“EMR can help in proper medication of patients,which is lacking at the moment”*.

Additionally, EMRS was seen as a tool to prevent the dispensing of expired medications from the pharmacy and increase patient compliance: *“It [EMRS] will prevent dispensing of expired medicines” *(P-28). There was also an expectation that EMRS could help control antibiotic resistance by supporting the analysis of treatment efficacy and monitoring follow-ups, thus avoiding overprescribing or the use of too broad-spectrum antibiotics. P-102 wrote, *“Resistance against antimicrobial drugs will be reduced”.*

#### Paperless and cost-effective

The transition to a paperless system was seen as an advantage, offering space-saving, and reducing the risk of data loss. P-47 described this benefit as, *“It is paperless*,* so will be cost-effective and will also protect against loss of data”.*

#### Improvement in the healthcare system

The implementation of EMRS was anticipated to enhance overall healthcare system quality. P-22 highlighted, *“Management control of quality monitoring may be easy”.* Participants mentioned improvements in quality monitoring and health facility operations. They also believed that EMRS could expedite service delivery and improve patient care by streamlining record handling: *“Easy handling of records will lead to getting better service to the patient (P-24)”*

#### Legal benefits

Increased transparency and accountability in medical record-keeping were considered benefits for law-related issues, *“It will provide more transparency and less burden of storage of patient data”* (P-72).

Overall, these responses highlighted the multifaceted advantages associated with the implementation of EMRS in healthcare facilities.

### Digitalization preference

**Fig. 3:**
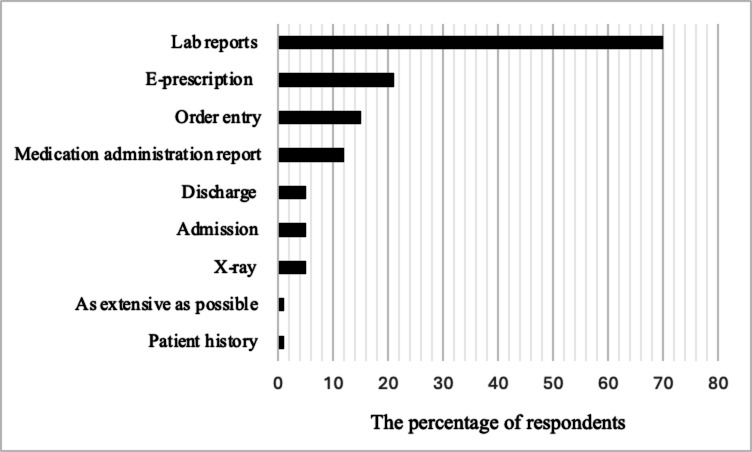
Respondents’ preferences for digitalizing routine work in the Facility (*N* = 73, 70%)

In response to the open-ended question regarding which parts of their routine work would they prefer for digitalization, (*n* = 73, 70%) the respondents expressed a variety of preferences (Fig. 3).

The majority (*n* = 51, 70%) indicated a desire for the digitalization of lab reports. Additionally, e-prescriptions emerged as another crucial aspect, with (*n* = 15, 21%) participants advocating for their digitalization.

## Discussion

This study provides valuable insights into EMRS implementation in an Indian private-sector hospital. It highlights the specific challenges and opportunities faced by an academic hospital in India, contributing to the broader understanding of EMRS in this context.

### Clinical training and perceptions of EMRS

A statistically significant association was found between the year of the postgraduate specialization program and the perceived usefulness of the ESS for prescribing medication, particularly among third-year students. It suggests that advancement in time of clinical training increases the recognition of the practical value of the ESS in clinical decision-making. It indicates that more experienced doctors who have encounters with more complex cases and clinical responsibilities might better appreciate the structured guidance provided by the ESS. Furthermore, 68% of respondents believed that an ESS would be beneficial for prescribing medication.

The participants, who are doctors and future specialists, will prescribe antibiotics and play a crucial role in antimicrobial resistance (AMR). Early exposure and training to use ESS and EMRS can promote responsible antibiotic-prescribing practices in the future. EMRS functionalities, such as prescription alerts and medication usage monitoring, can support doctors in making informed decisions, thereby promoting responsible antibiotic use [39–41]. This underscores the potential need for early integration of ESS and EMRS in medical education. However, the participants’ opinions on the potential time-saving advantages of ESS were divided. The mixed responses may reflect the diverse experiences and expectations of healthcare professionals regarding the implementation and effectiveness of these support systems within their practice and need further studies.

### Perceived barriers and challenges of EMRS implementation

Participants highlighted concerns about staff competency, inadequate training, and a lack of IT personnel. Additionally, 86% considered unreliable internet as a barrier to EMRS implementation, with 42% considering it a major challenge. A systematic review in the United States of America (USA) found cost, user perceptions, and implementation process to be key challenges, while facility characteristics and IT infrastructure were less common [42]. Another systematic review identified technological limitations as major barriers to India’s digital healthcare transformation [43].

Although specific research on internet access and EMR adoption in India is limited, strengthening internet infrastructure is crucial. Unlike LMICs, USA-based studies did not mention internet access, possibly assuming it as a basic part of the facility’s infrastructure [42]. This variation in perceived barriers between HICs and LMICs underscores the need for tailored strategies to overcome challenges in diverse healthcare settings.

Moreover, our study identified financial constraints as a key barrier, with 44% citing a lack of funds as a major barrier. This finding resonates with similar global studies, where financial limitations have been identified as a primary obstacle to EMRS adoption [44–47]. This emphasizes the need for strategic resource allocation and budget planning to support implementation efforts.

Organizational challenges, such as staff shortages and resistance, were universal barriers to EMRS adoption [44, 45, 47]. Regulatory obstacles and inadequate infrastructure were also frequently reported [44, 45, 47]. These factors further complicate the implementation process and emphasize the need for management strategies and staff engagement initiatives [28]. Organizational culture, including supportive leadership, open communication, comprehensive infrastructure planning, technological support and staff collaboration, significantly influences the success of EMRS adoption [48]. Exploring the role of organizational culture in shaping attitudes toward EMRS adoption warrants in-depth investigation, especially in resource-limited settings.

A multi-faceted approach is suggested to address the above-mentioned barriers and facilitate successful EMRS implementation in such settings. Financial limitations can be managed through strategic resource allocation, including prioritizing budget planning, seeking government grants, and exploring private partnerships [23, 28]. Conducting cost-benefit analyses to demonstrate potential savings and patient outcome improvements can further justify investments [45, 46]. Organizational challenges, such as staff resistance, can be addressed with effective change management plans, including leadership engagement to champion the EMRS initiative and involving staff in planning can increase acceptability and reduce resistance and fostering a user-centred design [45, 46]. Addressing these issues further requires targeted training programs to enhance staff competency, securing adequate funding and resources, and establishing robust technology and support systems. Regulatory obstacles require proactive compliance and advocacy. Staying updated with current regulations and investing in a robust IT infrastructure will support ongoing technical needs and compliance [45, 46].

### Perceived benefits of EMRS implementation

A majority of respondents expressed a notable positive attitude towards EMRS implementation (93%), featuring the recognition of the potential benefits of digitalization in healthcare delivery. Our findings indicate that less than half of the respondents (41%) believe that EMRS saves time. This aligns with insights from a multicentric qualitative study conducted among physicians and nurses in trauma hospitals, academic medical centres, medical clinics, home health centres, and small hospitals in the USA [49]. In another study conducted in Saudi Arabia, a HIC demonstrated that 34% of physicians agreed with the time-saving capacity of EMRS and 27% strongly agreed that EMRS could save time [50]. This discrepancy regarding the potential time-saving advantages of EMRS highlights the need for further exploration of the gains associated with EMRS adoption and clarification of expectations among stakeholders.

Moreover, our study participants expressed optimism about the potential transformative impact of EMRS on healthcare delivery. The majority anticipated improvements in the accessibility and retrieval of clinical data, enhanced management control, and streamlined healthcare processes. These anticipated benefits align with findings from studies conducted in the USA, which have highlighted the potential of EMRS to improve efficiency, patient safety, and overall quality of care [49]. Moreover, the emphasis on potential cost savings and time efficiency resonates with similar studies, indicating a consistent recognition of these benefits among healthcare professionals worldwide [51]. These anticipated benefits align with the overarching goals of EMRS adoption, underscoring the transformative potential of these systems in optimizing healthcare processes and outcomes [52].

### Digitalization preference

The unique aspect of this study lies in the specific question asked to participants, which focused on identifying particular areas of routine work they believed would benefit most from digitalization, offering a targeted understanding of their preferences and perceived priorities. Notably, the participants expressed a strong desire to prioritize the digitalization of lab reports, e-prescriptions, order entry processes, and medication administration. This finding underscores the importance of aligning digitalization efforts with staff needs and workflow requirements to maximize the potential benefits of EMRS implementation.

### Sustainability, frameworks, and future directions for EMRS implementation

Our study highlights the perceived barriers and benefits involved in adopting digital healthcare solutions and provides guidance for optimizing the EMRS implementation process. The sustained integration of EMRS can significantly enhance clinical decision-making and patient safety, particularly through features like electronic prescribing [16, 31]. Long-term benefits include improved data-driven decision making in patient care, enabling healthcare providers to make timely, informed decisions that improve patient outcomes [53]. Additionally, EMRS plays a vital role in monitoring antimicrobial use [53]. By tracking antibiotic prescriptions, it supports targeted antibiotic stewardship programs, helping to prevent over-prescription and combat AMR, a significant health threat [53].

The Precede-Proceed model is a systematic approach, where the problems are first identified, followed by exploring participants’ preparation and understanding, and then interventions are developed, introduced and evaluated [54, 55]. Following structured implementation frameworks is essential to ensure a systematic and effective EMRS adoption process, addressing key challenges and optimizing long-term sustainability.

Our study serves as a foundation of the primary phase focused on identifying key challenges and perceptions related to EMRS adoption. Preparing participants for EMRS implementation is critical and involves motivating healthcare professionals in a supportive manner rather than applying pressure [54, 55]. Our study adheres to this model and lays a strong foundation for successful intervention and sustainable long-term impact in subsequent stages. Based on the insights gained in this initial phase, future phases will include the development of tailored interventions to enhance acceptability and expand EMRS implementations.

Although this study focused on a tertiary care setting, the findings have potential implications for strengthening PHC [25]. Integrating PHC with tertiary care hospitals through EMRS can facilitate seamless data sharing and patient tracking across care levels. This is especially important in contexts like India, where patients often transit between PHC and higher-level facilities without comprehensive medical records, leading to repeated or inappropriate antibiotic prescriptions. Enhanced record continuity could significantly support antimicrobial stewardship efforts [56]. Recent PHC reforms in India, including the establishment of Health and Wellness Centres under the Ayushman Bharat program, provide a valuable opportunity to integrate EMRS into the PHC infrastructure and extend digital benefits to the primary level [57].

### Strengths and limitations

The study has several strengths that employ the use of a comprehensive questionnaire with closed- and open-ended questions, capturing various aspects of EMRS implementation. This approach highlights the perceived benefits and barriers among healthcare professionals in a private, rural, tertiary care facility in a LMIC. The validated questionnaire, developed through literature review and pilot testing, can be implemented in other similar settings after careful testing. Including participants from diverse departments and experience levels enhances the generalizability. However, limitations include its single centre design and small sample size, which may not fully represent all Indian healthcare facilities. Yet, many private-sector hospitals in India share similar operational structures and resources [58, 59], making these findings carefully transferable to a significant extent to a broader Indian private-sector context and other similar LMIC settings. To enhance generalizability in future research, stratified random sampling could help ensure broader representation across diverse healthcare settings.

Additionally, a self-reported questionnaire introduces the risk of response bias but remains a cost-effective method to record reflections from larger groups while maintaining the anonymity of the participants. However, self-administered questionnaires are also frequently used in exploratory studies, particularly for assessing perceptions and attitudes [60]. Lastly, while this study identifies key barriers and benefits, deeper contextual exploration is needed. Future research should integrate qualitative methods like focus groups, in-depth interviews, and detailed surveys for a more comprehensive understanding.

### Implications for healthcare practice and policy

The findings from this study offer valuable insights for healthcare facilities, practice and policy, especially regarding strategies to facilitate EMRS adoption in private hospitals in a LMIC. By displaying the perceived barriers such as financial limitations, staff resistance, and regulatory challenges, this study highlights key areas where targeted interventions are needed to improve the success of EMRS implementation. Healthcare facilities may benefit from investing in modifying management strategies and preparing the staff through training and building confidence for EMRS implementation. Moreover, our findings highlight the need for policymakers’ support to address financial and infrastructural barriers in private-sector facilities. They might consider subsidies, grants, encouragements, or incentives to promote EMRS adoption in low-resource settings, including private sector facilities.

## Conclusion

Our study provides important perspectives on the dynamics of EMRS implementation in an Indian private healthcare facility, specifically highlighting the perceived barriers, preparedness, and anticipated benefits among healthcare professionals and offers thoughtful considerations for both healthcare professionals and researchers. The participants recognized various barriers, such as financial constraints, insufficient infrastructure, technological and resource limitations and resistance to change. At the same time, the critical role of EMRS was also acknowledged in enhancing resource efficiency, patient care, and overall facility upscaling. Therefore, our study fills the key gap in understanding healthcare professionals’ perspectives on EMRS readiness and implementation in LMICs settings.

Furthermore, the findings lay the groundwork for evidence-based strategies to support successful EMRS implementation, emphasizing the need for structured frameworks that prepare and motivate healthcare professionals. To facilitate smoother adoption and maximize EMRS benefits, tailored intervention models should be explored. Policymakers and administrators must focus on targeted capacity-building initiatives, sustainable funding models, and a stepwise approach to EMRS implementation to address the key barriers. Additionally, this study provides a convincing rationale for expanding research on a larger scale to better understand the complexities of EMRS implementation in private healthcare facilities in LMICs. Further in-depth qualitative study will provide essential and actionable recommendations to optimize EMRS effectiveness, ultimately contributing to improved healthcare delivery and public health outcomes in LMICs.

## Supplementary Information

Supplementary Material 1.

## Data Availability

To protect participants’ privacy and maintain participants’ anonymity, the datasets generated and analysed during the current study cannot be shared publicly but can be made available upon suitable request.

## Abbreviations

AMR: Antimicrobial resistance
EMRS: Electronic medical record systems
ESS: Electronic support system
HICs: High-income countries
HMIS: Health management information systems
LMICs: Lower-middle-income countries
PHCs: Primary health centres
MBBS: Bachelor of Medicine and Bachelor of Surgery
MD: Doctor of Medicine
MS: Master of Surgery
IT: Information technology
USA: United States of America
