# Supplementary material for: Perceived benefits and barriers of medical doctors regarding electronic medical record systems in an Indian private-sector healthcare facility
Source: BMC Health Serv Res. 2025 May 19;25:719. doi: 10.1186/s12913-025-12877-5 (PMC12087044; doi:10.1186/s12913-025-12877-5)
Supplement: Supplementary file 1 — Supplementary Material 1. [file 12913_2025_12877_MOESM1_ESM.docx]

# **Perceived Benefits and barriers to implement Electronic Medical Record System (EMRS).**

# ***All responses and the respondent's details will be kept confidential.***

# **Respondent’s information: Date: _____________**

**Q. 1.** Sex: Male/Female. **Q. 2.** Year of postgraduate specialization: _______________________

**Q. 3.** Department: _____________________

**Q. 4. Do you think there is a need for the implementation of an EMRS in the facility? Yes / No**

**If yes, what benefits do you predict? ______________________________________________________________**

**Q.5. What do you think about having an electronic support system that can suggest best suited medicines to be prescribed, based on the complaints and diagnosis of the patients?**

a. It will be useful b. It will be impractical c. It will save time.

d. It will be useless e. Can’t say.

**Q. 6.  A. Which of the following data is regularly collected, analyzed, and presented in your setting, to assess the quality of healthcare?** **Please *Write ‘YES’ or ‘NO’ in front of each option***

1. Medication error reports
2. Medication and Treatment regimen to patients during hospitalization and follow-up treatment
3. Occupational hazards such as needle prick injury
4. Healthcare-Associated Infections report
5. Bed Occupancy rate
6. Incidence reports from wards to higher authorities
7. Patient records are analyzed regularly
8. Adverse drug reactions
9. Antibiotic Resistance patterns
10. Other (specify): ____________________________________________

**Q.6. B. Which of the following reports are available electronically in your setting? *Tick all applicable options***

1. Lab reports
2. Patients’ referral
3. Discharge
4. Consultancy
5. None
6. Other (please specify): _______________________________________________________________

***Perceived Barriers to Adopt & Use of Electronic Medical Records* System *(EMRS)*** *at your facility/ Department.*

**Q. 7.  Keeping in mind-** the implementation of an EMR system in the setting, indicate which factors you think are **A= Major Barrier, B=Possible to manage and C= Not Applicable**

- **Financial issues**

| **Factors** | **Mark A/B/C** | **Factors** | **Mark A/B/C** | **Factors** | **Mark A/B/C** |
| --- | --- | --- | --- | --- | --- |
| Lack of funds |  | Capital investment |  | - |  |
| - **Organizational Issues** | | | | | |
| Time management |  | Lack of Infrastructure |  |  |  |
| Lack of staff |  | Staff Resistance |  | Lack of IT personnel |  |
| Staff competency |  | Lack of coordination |  | Possible loss of productivity during transition of records |  |
| Changeover of information during transition of records |  | Lack of trainer to train staff about EMR system |  | Lack of management support |  |
| - **Legal or Regulatory Issues** | | | | | |
| Perceived concerns for confidentiality breaches |  | Laws & regulations for electronic signatures. How to validate? |  | Not sure about jurisdiction acceptability of EMRS |  |
| - **Technological Issues** | | | | | |
| Finding a system that meets the needs of the facility |  | Concerns that the system will become obsolete/ outdated |  | Wireless or internet access |  |
| Software or hardware incompatibilities with the established systems |  | Training to all levels of staff |  | - |  |

**Q. 8. Any other factors that you consider as barriers for digitalizing the Medical Records in your facility (EMRS)?**

_______________________________________________________________________________________

_______________________________________________________________________________________

_______________________________________________________________________________________

***Perceived* benefits *to Adopt & Use of Electronic Medical Records* System *(EMRS)*** *at your facility/ Department.*

**Q. 9.** Indicate which factors that you recognize to be **a major or minor benefit, or not a Benefit but is a *Barrier*** for the implementation of an EMR system to support clinical work functions.

**Write A / B/ C: where, A=Major Benefit, B=Minor Benefit and C=This is not a Benefit but a *Barrier***

| **Factors** | | **Major Benefit** **(A)** | **Minor** **Benefit** **(B)** | **This is a *Barrier* (C)** |
| --- | --- | --- | --- | --- |
| a | Anyone from anywhere, at any time, can access to clinical data (by multiple users, from multiple locations) |  |  |  |
| b | Management control will be easier |  |  |  |
| c | Quality monitoring will be easy |  |  |  |
| d | Enhanced efficiency due to digital records |  |  |  |
| e | Staff will be empowered, and staff satisfaction will be obtained |  |  |  |
| f | Attractive job feature when recruiting new staff |  |  |  |
| g | In computer programs the information entered at one point can be seen on other screens as well, this will save time i.e., faster, and more accurate billing with integrated data systems |  |  |  |
| h | Improved regulatory of treatment compliance |  |  |  |
| i | Ability to electronically exchange data with other providers hospitals, medical offices, labs, pharmacies: faster exchange |  |  |  |
| j | Cost savings and time saving |  |  |  |
| k | Patient safety (minimum medical errors due to handwriting error like- like and sound like) |  |  |  |
| l | Treatment planning can be improved as all information will be available at one point |  |  |  |
| m | Improved communication within the facility: among staff between shifts, labs, and various wards |  |  |  |

**Q. 10. Any other expected benefits of electronic medical records System (EMRS)?**

____________________________________________________________________________________________

____________________________________________________________________________________________

**Q. 11. Which parts of your routine work do you want to be digitalized? (**Such as order entry, e-prescribing, Medication Administration Record, Receive lab reports via digital network to the wards**)**

____________________________________________________________________________________________

____________________________________________________________________________________________

**Q. 12. Do you think that the implementation of an EMRS will be successful in your setting? Yes/No**

*Thank you for your time!*
